# Association of Affective Temperaments With One‐Year Depressive Outcomes in Middle‐Aged and Older Patients With Mood Disorders Without Psychiatric Comorbidities

**DOI:** 10.1002/npr2.70170

**Published:** 2026-09-16

**Authors:** Takashi Ozaki, Hisashi Narita, Shizuka Mito, Niki Udo, Nobuyuki Mitsui, Naoki Hashimoto, Ichiro Kusumi, Takahiro A. Kato

**Affiliations:** ^1^ Department of Psychiatry, Graduate School of Medicine Hokkaido University Sapporo Hokkaido Japan; ^2^ Ishikari Familia Hospital Sapporo Hokkaido Japan; ^3^ Sapporo Suzuki Hospital Sapporo Hokkaido Japan

**Keywords:** affective temperament, bipolar disorder, comorbidity, major depressive disorder, subsyndromal

## Abstract

**Aim:**

The course of depressive symptoms in mood disorders may be influenced by multiple clinical factors, making predictors of subsequent symptoms difficult to identify. This study examined whether affective temperaments are associated with depressive symptoms at one‐year follow‐up in middle‐aged and older patients with mood disorders without clinically relevant psychiatric comorbidities.

**Methods:**

This one‐year longitudinal observational study included 91 patients aged 40–75 years with major depressive disorder (MDD) or bipolar disorder (BD) who were admitted to Hokkaido University Hospital for a recent major depressive episode, completed baseline assessments at discharge, and were followed for 1 year after discharge. Patients with clinically relevant psychiatric comorbidities were excluded. Affective temperaments were assessed using the Temperament Evaluation of Memphis, Pisa, Paris, and San Diego Autoquestionnaire. Multiple linear regression analyses were performed to examine factors associated with the 17‐item Hamilton Depression Rating Scale (HAM‐D17) total score at one‐year follow‐up, and logistic regression analyses to examine factors associated with subthreshold or more severe depressive symptoms at one‐year follow‐up. Temperament‐by‐diagnosis interaction analyses were performed to examine whether these associations differed between MDD and BD. This study was approved by the ethics committee of Hokkaido University Hospital.

**Results:**

Depressive and cyclothymic temperaments were significantly associated with a higher HAM‐D17 total score at one‐year follow‐up after adjustment. Depressive temperament was also associated with non‐remission. A significant cyclothymic temperament‐by‐diagnosis interaction was observed.

**Conclusion:**

Depressive and cyclothymic temperaments may be clinically relevant for understanding residual depressive symptoms at one‐year follow‐up in middle‐aged and older patients with mood disorders without clinically relevant psychiatric comorbidities. The significant cyclothymic temperament‐by‐diagnosis interaction highlights the potential diagnosis‐specific relevance of affective temperament. Larger prospective studies are required to confirm these findings.

## Abstract 2

Depressive and cyclothymic temperaments were associated with greater depressive symptom severity at one‐year follow‐up in patients with mood disorders. Depressive temperament was also associated with non‐remission, while the association between cyclothymic temperament and depressive symptoms differed between MDD and BD.

## Graphical abstract

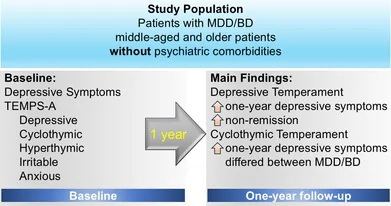

## 1 Introduction

The longitudinal course of depressive symptoms in mood disorders among patients with major depressive disorder (MDD) or bipolar disorder (BD) is influenced by multiple clinical factors and cannot be fully explained by diagnostic category alone [1, 2, 3, 4]. Depression is a heterogeneous condition, and its clinical course may be affected by illness characteristics, treatment response, physical health, functional status, cognitive factors, psychosocial factors, and age‐related changes [5, 6]. Psychiatric comorbidities, such as comorbid anxiety, may further affect depressive outcomes and are reportedly associated with a more unfavorable course of depression and lower remission rates in patients with MDD and BD [7, 8, 9, 10, 11]. Age‐related factors are also important. In older patients, depression may be influenced by features such as cognitive changes, vascular burden, and physical comorbidities [6, 12, 13, 14, 15]. A previous study targeting middle‐aged adults predicted future depressive symptoms by using a relatively small number of factors [15], suggesting that focusing on a clinically defined sample without clinically relevant psychiatric comorbidities and with a limited age range may be useful for identifying factors associated with subsequent depressive symptoms [10]. In addition, previous reports concerning the structure of depressive symptoms suggest that, in cases of mild depression in the general population, individual depressive symptoms behave in a complex and non‐linear manner [16]. In contrast, studies focused on late‐life depressive symptoms have shown that these symptoms can be attributed to a limited number of latent variables [17], confirming that limiting the target population can play an important role in understanding the characteristics of depressive symptoms.

When identifying factors associated with depressive symptoms post‐diagnosis, affective temperaments may provide useful frameworks for evaluating mood‐related vulnerability that is not fully captured by categorical diagnosis. Affective temperaments refer to relatively stable patterns of mood reactivity and emotional regulation. The Temperament Evaluation of Memphis, Pisa, Paris, and San Diego Autoquestionnaire (TEMPS‐A) was developed to assess depressive, cyclothymic, hyperthymic, irritable, and anxious temperaments [18]. Previous studies have linked affective temperaments, particularly depressive and cyclothymic temperaments, to diagnostic differentiation, treatment difficulty, bipolar‐spectrum pathology, suicidality, and depressive treatment response [19, 20, 21, 22, 23]. However, evidence remains limited regarding whether these temperaments are associated with subsequent depressive symptoms in clinically defined longitudinal samples with restricted age range and without clinically relevant psychiatric comorbidities.

Bipolar‐spectrum features are also considered conventional clinical indicators to characterize latent bipolarity among patients presenting with depressive episodes. Recurrent depressive episodes, early onset, treatment‐resistant depression, antidepressant‐induced hypomania or mania, mixed depressive features, family history of BD, and subthreshold manic symptoms have been reported to be clinical indicators of bipolarity [24, 25, 26, 27, 28, 29, 30, 31, 32, 33]. However, evidence remains mixed regarding whether bipolar‐spectrum features consistently explain poor depressive outcomes. In STAR*D, a landmark U.S. effectiveness trial of antidepressant treatment for MDD, bipolar‐spectrum features did not substantially explain antidepressant treatment resistance [34]. Therefore, while bipolar‐spectrum features are clinically important for diagnostic formulation and the assessment of latent bipolarity, their relationship with subsequent depressive symptoms remains unclear.

The present study aimed to investigate whether affective temperaments are associated with depressive symptoms at one‐year follow‐up in middle‐aged and older patients with mood disorders without clinically relevant psychiatric comorbidities. We included patients with MDD and BD because both disorders can present with major depressive episodes, and residual depressive symptoms after such episodes are clinically important across diagnostic categories. Diagnostic group was included as a covariate in the main analyses, and temperament‐by‐diagnosis interaction analyses were performed to examine whether the associations between affective temperaments and depressive symptoms differed between MDD and BD.

## 2 Methods

### 2.1 Study Design and Participants

This was a one‐year longitudinal observational study conducted at Hokkaido University Hospital between July 2013 and July 2023. Among patients admitted to the Department of Psychiatry during this period, those who had experienced a major depressive episode within 3 months before baseline (i.e., discharge) and were diagnosed with MDD, bipolar I disorder, or bipolar II disorder were considered for inclusion. Patients were included in the present analysis if they were aged 40–75 years, provided written informed consent, completed the baseline assessment at discharge, were followed for 1 year after discharge, and retained the same diagnosis of MDD or BD throughout the one‐year follow‐up period. After discharge, treatment was continued on an outpatient basis, and patients completed the one‐year follow‐up assessment. Diagnoses were made according to the Diagnostic and Statistical Manual of Mental Disorders, Fifth Edition [35]. Diagnostic assessments were performed by experienced psychiatrists with more than 10 years of clinical experience.

Patients with serious physical illnesses or other organic conditions were excluded. Patients with clinically relevant psychiatric comorbidities, including anxiety disorders, obsessive‐compulsive disorder, phobias, substance use disorders, personality disorders, schizophrenia spectrum disorders, neurodevelopmental disorders, eating disorders, intellectual disabilities, and dementia, were also excluded.

### 2.2 Clinical Assessments

The baseline assessment was performed at discharge from the index hospitalization for a recent major depressive episode. At baseline, depressive symptoms, manic symptoms, bipolar‐spectrum features, affective temperaments, psychotropic medication use, and demographic and clinical variables were assessed. Depressive symptoms were assessed using the 17‐item Hamilton Depression Rating Scale (HAM‐D17) [36], and manic symptoms were assessed using the Young Mania Rating Scale (YMRS) [37]. At one‐year follow‐up, depressive symptoms were reassessed using the HAM‐D17.

### 2.3 Affective Temperaments

Affective temperaments were assessed at baseline using the TEMPS‐A, which assesses five affective temperaments: depressive, cyclothymic, hyperthymic, irritable, and anxious temperaments [38]. Each item was scored dichotomously, and each temperament score was calculated as the mean score of items belonging to the corresponding subscale [18, 39]. Each temperament score was treated as a continuous variable.

### 2.4 Bipolar‐Spectrum Features

As exploratory analyses, associations between selected bipolar‐spectrum features and depressive symptoms at one‐year follow‐up were examined. Based on previously reported clinical features suggestive of bipolarity, the examined features included recurrent major depressive episodes, defined as three or more lifetime major depressive episodes; early onset of mood disorder, defined as onset before 25 years of age; treatment‐resistant depression, defined as nonresponse to at least three adequate antidepressant trials; a history of antidepressant‐induced hypomania or mania; mixed depressive features according to Benazzi's criteria; and baseline Young Mania Rating Scale (YMRS) score [26, 27, 28, 34, 40]. Mixed depressive features were assessed according to Benazzi's criteria, based on previous work suggesting that these criteria may capture bipolar‐spectrum manifestations during major depressive episodes more sensitively than the DSM‐5 mixed‐features specifier [41]. In addition, family history of BD in first‐degree relatives, history of psychotic depression, and history of postpartum depression were assessed as potential bipolar‐spectrum features. However, these variables were excluded from subsequent analyses because the number of participants with each feature was less than 5% of the total sample, which could lead to unstable estimates.

### 2.5 Outcomes

The main outcome was HAM‐D17 total score at one‐year follow‐up. Depressive symptoms were assessed using HAM‐D17 at baseline and at one‐year follow‐up. Remission was defined as a HAM‐D17 total score of 7 or lower, and non‐remission as a HAM‐D17 total score of 8 or higher. Subthreshold depressive symptoms were defined as a HAM‐D17 total score of 8–14 [42]. As an exploratory supplementary outcome, the suicidal ideation item of the HAM‐D17 at one‐year follow‐up was also examined.

### 2.6 Statistical Analysis

The main analyses were performed with the combined MDD and BD sample because the study focused on depressive symptoms after a recent major depressive episode across diagnostic categories. Diagnostic group was included as a covariate in these models, and temperament‐by‐diagnosis interaction analyses were performed as exploratory analyses.

Baseline characteristics were summarized for the total sample and by diagnostic group. Continuous variables are presented as means and standard deviations, and categorical variables as numbers and percentages. Differences between patients with MDD and BD were examined using Welch's *t*‐test for continuous variables and the chi‐square test for categorical variables. When sample sizes were small, data were assessed using the Mann–Whitney *U* test for continuous variables and Fisher's exact test for categorical variables.

Correlations among the five TEMPS‐A temperament scores were examined using Spearman's rank correlation coefficients and are presented in Table S1.

Multiple linear regression analyses were performed to examine associations between TEMPS‐A affective temperaments and HAM‐D17 total score at one‐year follow‐up, and between bipolar‐spectrum features and HAM‐D17 total score at one‐year follow‐up. Each temperament (or bipolar‐spectrum feature for the latter) was entered separately into the model. The models were adjusted for age, sex, diagnostic group, baseline HAM‐D17 total score, and use of psychotropic medications. Psychotropic medication use was included in the regression models as four separate binary covariates: antidepressant use, mood stabilizer use, antipsychotic use, and benzodiazepine use. Exploratory multiple linear regression analyses were also performed to examine associations between TEMPS‐A affective temperament scores and the suicidal ideation item of HAM‐D17 at one‐year follow‐up. Each temperament score was entered separately into the model. Binary logistic regression analyses were performed to examine associations between TEMPS‐A affective temperaments and non‐remission at one‐year follow‐up. Each temperament was entered separately into the model. For these analyses, models were adjusted for age, sex, diagnostic group, baseline HAM‐D17 total score, and use of psychotropic medications, including antidepressants, mood stabilizers, antipsychotics, and benzodiazepines.

To examine whether the associations between affective temperaments and depressive symptoms differed according to diagnostic group, separate temperament‐by‐diagnosis interaction models were fitted for each TEMPS‐A dimension. Each model included age, sex, diagnosis, baseline HAM‐D17 total score, antidepressant use, mood stabilizer use, antipsychotic use, benzodiazepine use, the corresponding standardized TEMPS‐A temperament score, and the temperament‐by‐diagnosis interaction term. The one‐year HAM‐D17 total score was standardized. MDD was used as the reference category for diagnosis.

As a sensitivity analysis, subsequent diagnostic information available after the predefined one‐year study period was reviewed for patients diagnosed with MDD at baseline. Patients who were subsequently reclassified as having BD were reassigned, and the primary linear regression analyses and temperament‐by‐diagnosis interaction analyses were repeated using the revised diagnostic classification.

All statistical analyses were performed using R version 4.5.1 [43]. *p* < 0.05 was considered statistically significant.

## 3 Results

### 3.1 Participant Characteristics

The final sample consisted of 91 patients, including 47 patients with MDD and 44 patients with BD. Among patients with BD, 21 had bipolar I disorder and 23 had bipolar II disorder. The mean age of the total sample was 53.1 years, and 47 patients were female. At baseline, the mean HAM‐D17 total score was 6.5 and the mean YMRS total score was 1.36. At one‐year follow‐up, the mean HAM‐D17 total score was 9.1. Thirty‐nine (42.9%) patients were classified as having remission, 35 (38.5%) as having subthreshold depressive symptoms, and 17 (18.7%) as having depressive symptoms (Table 1).

**TABLE 1:** Baseline characteristics of participants with major depressive disorder (MDD) and bipolar disorder (BD).

Variables	Total (*n* = 91)	MDD (*n* = 47)	BP (*n* = 44)	*p*
Age, Mean, SD	53.1, 11.2	53.6, 12.9	52.6, 9.0	0.956
Sex				0.144
Male, *n*, %	44, 48.4	19, 40.4	25, 56.8	
Female, *n*, %	47, 51.6	28, 59.6	19, 43.2	
Clinical Scores (Baseline)				
HAM‐D17 total score, Mean, SD	6.5, 5.1	7.1, 4.8	5.9, 5.5	0.105
HAM‐D17 suicidal ideation item score, Mean, SD	0.2, 0.5	0.3, 0.5	0.2, 0.5	0.213
YMRS total score, Mean, SD	1.4, 2.4	0.7, 1.5	2.1, 3.0	0.005*
Clinical Scores (1‐year follow‐up)				
HAM‐D17 total score, Mean, SD	9.1, 6.7	8.8, 7.2	9.5, 6.3	0.413
HAM‐D17 suicidal ideation item score, Mean, SD	0.5, 0.8	0.5, 0.8	0.5, 0.9	0.886
Remission state, *n*, %	39, 42.9	23, 48.9	16, 36.4	
Subsyndromal state, *n*, %	35, 38.5	14, 29.8	21, 47.7	
Depressive state, *n*, %	17, 18.7	10, 21.3	7, 15.9	
Bipolar‐spectrum features				
Recurrent MDEs, *n*, %	35, 38.5	10, 21.3	25, 56.8	< 0.001**
Early age at onset of first MDE, *n*, %	8, 8.8	1, 2.1	7, 15.9	0.027*
Treatment‐resistant depression, *n*, %	26, 28.6	12, 25.5	14, 31.8	0.643
Antidepressant‐induced hypomania or mania, *n*, %	27, 29.7	5, 10.6	22, 50.0	< 0.001**
Mixed features according to Benazzi's criteria, *n*, %	13, 14.3	0, 0.0	13, 29.5	< 0.001**
Affective temperament				
Depressive temperament, Mean, SD	1.5, 0.2	1.5, 0.2	1.5, 0.2	0.424
Cyclothymic temperament, Mean, SD	1.3, 0.2	1.3, 0.2	1.3, 0.3	0.561
Hyperthymic temperament, Mean, SD	1.2, 0.2	1.2, 0.2	1.2, 0.3	0.608
Irritable temperament, Mean, SD	1.2, 0.2	1.2, 0.2	1.2, 0.2	0.917
Anxious temperament, Mean, SD	1.4, 0.3	1.5, 0.3	1.4, 0.3	0.363
Psychotropic drugs use				
Antidepressant use, *n*, %	43, 47.3	36, 76.6	7, 15.9	< 0.001**
Mood stabilizer use, *n*, %	49, 53.8	16, 34.0	33, 75.0	< 0.001**
Antipsychotic use, *n*, %	62, 68.1	25, 53.2	37, 84.1	0.002*
Benzodiazepine use, *n*, %	64, 70.3	33, 70.2	31, 70.5	1.000

Abbreviations: BD, bipolar disorder; HAM‐D17, 17‐item Hamilton Depression Rating Scale; MDD, major depressive disorder; MDE, major depressive episode; TEMPS‐A, Temperament Evaluation of Memphis, Pisa, Paris, and San Diego Autoquestionnaire; YMRS, Young Mania Rating Scale.

* *p* < 0.05.

** *p* < 0.001.

In addition, after the predefined one‐year follow‐up study period, 5 of the 47 patients initially diagnosed with MDD were reclassified as having BD. The median time to diagnostic conversion was 20.0 months (IQR, 18.0–27.0; range, 13.0–44.0 months).

### 3.2 Associations Between Affective Temperaments and Depressive Symptoms at One‐Year Follow‐Up

Table 2 shows the results of multiple linear regression analyses examining associations between TEMPS‐A affective temperaments and HAM‐D17 total score at one‐year follow‐up. Depressive temperament was significantly associated with a higher HAM‐D17 total score at one‐year follow‐up (standardized *β* = 0.21, 95% confidence interval 0.01 to 0.41, *p* = 0.037). Cyclothymic temperament was also significantly associated with a higher HAM‐D17 total score at one‐year follow‐up (standardized *β* = 0.22, 95% confidence interval 0.03 to 0.41, *p* = 0.022).

**TABLE 2:** Associations between affective temperaments and depressive symptoms at one‐year follow‐up.

Variables	*β*	95% CI	*p*	Adjusted *R* ^2^
Depressive temperament	0.21	[0.01, 0.41]	0.037*	0.28
Cyclothymic temperament	0.22	[0.03, 0.41]	0.022*	0.29
Hyperthymic temperament	0.16	[−0.03, 0.35]	0.096	0.27
Irritable temperament	0.13	[−0.06, 0.32]	0.190	0.26
Anxious temperament	0.20	[−0.01, 0.41]	0.059	0.28

*Note:* Separate multiple linear regression models were fitted for each TEMPS‐A temperament dimension. Standardized β coefficients and corresponding 95% confidence intervals were presented.

Abbreviations: *β*, Standardized *β*; CI, confidence interval; HAM‐D17, 17‐item Hamilton Depression Rating Scale; TEMPS‐A, Temperament Evaluation of Memphis, Pisa, Paris, and San Diego Autoquestionnaire.

* *p* < 0.05.

Anxious temperament showed a positive trend with HAM‐D17 total score at one‐year follow‐up (standardized *β* = 0.20, 95% confidence interval −0.01 to 0.41, *p* = 0.059). Hyperthymic temperament and irritable temperament were not significantly associated with HAM‐D17 total score at one‐year follow‐up.

In supplementary analyses, the depressive, cyclothymic, irritable, and anxious temperament scores were moderately to strongly intercorrelated (Spearman's *ρ* = 0.51 to 0.73, all *p* < 0.001) (Table S1).

In addition, cyclothymic temperament, irritable temperament, and anxious temperament were significantly associated with the suicidal ideation item of the HAM‐D17 at one‐year follow‐up (cyclothymic: standardized *β* = 0.27, 95% confidence interval = 0.05 to 0.49, *p* = 0.015; irritable: standardized β = 0.27, 95% confidence interval = 0.05 to 0.48, *p* = 0.018; anxious: standardized *β* = 0.27, 95% confidence interval = 0.03 to 0.50, *p* = 0.026). Depressive temperament showed a positive trend (standardized *β* = 0.18, 95% confidence interval = −0.04 to 0.41, *p* = 0.106), whereas hyperthymic temperament was not significantly associated with the suicidal ideation item (Table S2).

### 3.3 Associations Between Affective Temperaments and Non‐Remission at One‐Year Follow‐Up

Table 3 shows the results of binary logistic regression analyses examining associations between TEMPS‐A affective temperaments and non‐remission at one‐year follow‐up. Depressive temperament was significantly associated with non‐remission at one‐year follow‐up (adjusted odds ratio = 12.9, 95% confidence interval 1.2 to 170.2, *p* = 0.043), although the 95% confidence interval was wide. Cyclothymic, hyperthymic, irritable, and anxious temperaments were not significantly associated with non‐remission at one‐year follow‐up.

**TABLE 3:** Associations between affective temperaments and non‐remission at one‐year follow‐up.

TEMPS‐A	AOR	95% CI	*p*
Depressive temperament	12.9	[1.2, 170.2]	0.043*
Cyclothymic temperament	1.4	[0.2, 11.3]	0.728
Hyperthymic temperament	2.1	[0.2, 22.0]	0.522
Irritable temperament	2.3	[0.2, 33.3]	0.542
Anxious temperament	2.8	[0.4, 23.7]	0.340

Abbreviations: AOR, Adjusted odds ratio; CI, confidence interval; HAM‐D17, 17‐item Hamilton Depression Rating Scale; TEMPS‐A, Temperament Evaluation of Memphis, Pisa, Paris, and San Diego Autoquestionnaire.

* *p* < 0.05.

### 3.4 Interaction Between Temperaments and Diagnostic Group in Relation to Depressive Symptoms at One‐Year Follow‐Up

In the temperament‐by‐diagnosis interaction analyses, a significant interaction was observed only for cyclothymic temperament (standardized *β* = 0.45, 95% CI = 0.09 to 0.82, *p* = 0.015; Table 4 and Figure S1). No significant interactions were observed for the other temperament dimensions.

**TABLE 4:** Associations between affective temperaments and depressive symptoms at one‐year follow‐up according to diagnostic group.

Variables	HAM‐D17 total score at one‐year follow‐up			
	*β*	95% CI	*p*	Adjusted *R* ^2^
Depressive temperament				
TEMPS‐A (Depressive)	0.18	[−0.08, 0.44]	0.184	0.27
Diagnosis (BD vs. MDD)	−0.09	[−0.56, 0.38]	0.707	
TEMPS‐A × Diagnosis	0.08	[−0.30, 0.46]	0.684	
Cyclothymic temperament				0.33
TEMPS‐A (Cyclothymic)	−0.06	[−0.35, 0.23]	0.670	
Diagnosis (BD vs. MDD)	−0.16	[−0.62, 0.30]	0.482	
TEMPS‐A × Diagnosis	0.45	[0.09, 0.82]	0.015*	
Hyperthymic temperament				0.27
TEMPS‐A (Hyperthymic)	0.30	[−0.03, 0.63]	0.070	
Diagnosis (BD vs. MDD)	−0.07	[−0.55, 0.41]	0.770	
TEMPS‐A × Diagnosis	−0.22	[−0.62, 0.19]	0.293	
Irritable temperament				0.27
TEMPS‐A (Irritable)	−0.03	[−0.34, 0.27]	0.824	
Diagnosis (BD vs. MDD)	−0.07	[−0.55, 0.40]	0.755	
TEMPS‐A × Diagnosis	0.27	[−0.12, 0.65]	0.176	
Anxious temperament				
TEMPS‐A (Anxious)	0.05	[−0.24, 0.34]	0.725	0.28
Diagnosis (BD vs. MDD)	−0.07	[−0.54, 0.40]	0.776	
TEMPS‐A × Diagnosis	0.27	[−0.11, 0.65]	0.155	

*Note:* Multiple linear regression analyses were performed using standardized TEMPS‐A temperament scores and standardized HAM‐D17 total score at one‐year follow‐up. MDD was used as the reference category for diagnosis. The TEMPS‐A coefficient represents the association between each standardized temperament score and the standardized HAM‐D17 total score in the MDD group. The TEMPS‐A × diagnosis coefficient represents the difference in the TEMPS‐A slope between BD and MDD.

Abbreviations: *β*, Standardized *β*; BD, bipolar disorder; CI, confidence interval; HAM‐D17, 17‐item Hamilton Depression Rating Scale; MDD, major depressive disorder; TEMPS‐A, Temperament Evaluation of Memphis, Pisa, Paris, and San Diego Autoquestionnaire.

* *p* < 0.05.

### 3.5 Supplementary Analyses: Associations Between Bipolar‐Spectrum Features and Depressive Symptoms at One‐Year Follow‐Up

Exploratory analyses of bipolar‐spectrum features are presented in Table S3. None of the examined bipolar‐spectrum features showed a statistically significant association with HAM‐D17 total score at one‐year follow‐up.

### 3.6 Sensitivity Analysis of the Main Outcomes

In a sensitivity analysis, five patients subsequently reclassified from MDD to BD were reassigned according to their final diagnosis, and the main findings remained unchanged. Detailed results are shown in Tables S4 and S5.

## 4 Discussion

The present study examined whether affective temperaments are associated with depressive symptoms at one‐year follow‐up in middle‐aged and older patients with MDD or BD without clinically relevant psychiatric comorbidities. The main finding was that depressive and cyclothymic temperaments were associated with a higher HAM‐D17 total score at one‐year follow‐up. In addition, several temperaments were significantly associated with the suicidal ideation item of the HAM‐D17 at one‐year follow‐up. Depressive temperament was associated with non‐remission at one‐year follow‐up. A significant cyclothymic temperament‐by‐diagnosis interaction was also observed, indicating that the association between cyclothymic temperament and one‐year depressive symptom severity differed between MDD and BD. In addition, bipolar‐spectrum features examined in this study were not significantly associated with the HAM‐D17 total score at one‐year follow‐up. These findings suggest that affective temperaments may be clinically relevant for understanding subsequent depressive symptoms after a major depressive episode in middle‐aged and older patients with mood disorders. Although the association between affective temperaments and depressive symptoms may not be as unexpected as previously reported among heterogeneous samples with depression [18, 19], the present findings strengthen the previous findings by examining this association in a more clinically‐defined longitudinal sample with a restricted age range and without clinically relevant psychiatric comorbidities.

Affective temperaments may reflect stable patterns of mood reactivity and emotional regulation that are not fully captured by categorical diagnosis. Previous studies using TEMPS‐A have shown that affective temperaments are associated with clinical features of mood disorders, including diagnostic differentiation, treatment difficulty, bipolarity, and treatment response. In particular, depressive temperament is reported to be associated with poorer improvement in depressive symptoms in patients with mood disorders [19]. It is also noteworthy that a previous study targeting only a middle‐aged general population used a machine‐learning approach to predict the future onset of depression by using parameters including the frequency of depressed mood and personality‐related variables, such as the neuroticism score [15]. Such reports partially support our result demonstrating that depressive temperament was associated with higher depressive symptom severity at one‐year follow‐up among a strictly‐defined sample. This finding is of clinical importance because depressive temperament may be a clinically relevant marker of subsequent depressive symptoms even in middle‐aged and older patients with mood disorders without clinically relevant psychiatric comorbidities.

In exploratory supplementary analyses, cyclothymic, irritable, and anxious temperaments were associated with the suicidal ideation item at one‐year follow‐up. This finding is partially consistent with a previous study targeting a general Japanese adult population showing that depressive, irritable, and anxious temperaments were associated with suicide‐related ideations, although differences in sample characteristics, outcome assessment, and adjustment factors may explain the pattern of associations which differed from that of the present study [44].

Cyclothymic temperament is closely linked to the diagnosis of mood disorders. Previous studies have reported that cyclothymic temperament is associated with difficult‐to‐treat depression, diagnostic differentiation between MDD and BD, later bipolarity, and suicidality [20, 21, 22]. A large outpatient study also reported that cyclothymic temperament was strongly associated with bipolar II disorder [23]. In the present study, the significant cyclothymic temperament‐by‐diagnosis interaction suggests that the association between cyclothymic temperament and subsequent depressive symptom severity may differ according to diagnosis, as illustrated in Figure S1. This finding may indicate particular clinical relevance of cyclothymic temperament in BD and treatment difficulty, although confirmation in larger samples is needed.

In the present study, depressive temperament was associated with non‐remission at one‐year follow‐up. This finding is clinically important because non‐remission included subthreshold or more severe depressive symptoms. Previous studies have shown that residual depressive symptoms are associated with relapse, functional impairment, and poorer quality of life in patients with mood disorders [42, 45, 46, 47]. Therefore, depressive temperament may be relevant not only to symptom severity assessed as a continuous outcome, but also to clinically meaningful residual depressive burden. However, the association between depressive temperament and non‐remission should be interpreted with caution because the confidence interval was wide and the sample size was small.

Developmental factors, including childhood adversity, may influence both affective temperaments and subsequent depressive symptoms [48]. Because such factors were not assessed in the present study, their potential contribution should be examined in future studies.

In exploratory analyses, we also examined associations between bipolar‐spectrum features and depressive symptoms. These features were evaluated for distinguishing MDD from BD [24, 25, 26, 27, 28]. However, our results demonstrated no clear association with depressive symptoms at one‐year follow‐up in the present sample.

This study has several limitations. First, the sample size was small and present findings should be interpreted with caution and confirmed in a larger sample population, although several studies have used samples of similar size [10, 11]. In addition, because the main analyses were performed in the combined MDD and BD sample, diagnosis‐specific associations may not have been fully captured, although formal temperament‐by‐diagnosis interaction analyses were performed. Second, some patients initially diagnosed with MDD were subsequently reclassified as having BD during longer‐term follow‐up. However, the main findings remained unchanged after the sensitivity analysis. Third, because multiple statistical tests were performed, the present findings should be interpreted as exploratory. Fourth, TEMPS‐A scores may have been influenced by the recent major depressive episode, although TEMPS‐A was developed to assess relatively stable affective temperaments and previous validation studies have reported good test–retest reliability [49]. Fifth, because the TEMPS‐A temperament dimensions were intercorrelated, particularly depressive, cyclothymic, irritable, and anxious temperaments, and each temperament score was entered separately into the regression models, the observed associations cannot be interpreted as effects independent of the other temperament dimensions. Sixth, although patients with dementia and clinically apparent organic conditions were excluded, detailed measures of cognitive function, vascular burden, severity of physical comorbidities, and psychosocial functioning were not included. Seventh, excluding patients with clinically relevant psychiatric comorbidities may have reduced clinical heterogeneity and improved internal validity, but it may also limit the generalizability of the findings to routine clinical populations in which psychiatric comorbidities are common. Eighth, this was an observational study conducted at a single university hospital, and therefore the findings may not be generalizable to broader clinical populations, including patients who receive less intensive follow‐up. Finally, although psychotropic medication use was adjusted for as a covariate, medication use itself may reflect illness severity, treatment resistance, and other aspects of clinical complexity. Therefore, it may not always be regarded as a simple confounder. Further studies will be needed to determine whether the present findings can be replicated in more stratified clinical samples.

Despite these limitations, the present study suggests that affective temperaments assessed using TEMPS‐A, particularly depressive and cyclothymic temperaments, may be associated with residual depressive symptoms at one‐year follow‐up in middle‐aged and older patients with mood disorders without clinically relevant psychiatric comorbidities. Anxious temperament may also be relevant, although its association was not statistically significant. Future prospective studies with larger samples will be needed to evaluate a broader range of bipolar‐spectrum features, developmental information, cognitive and physical health measures, medication course, biological markers, and functional outcomes.

## 5 Conclusion

In this one‐year longitudinal observational study of middle‐aged and older patients with mood disorders without clinically relevant psychiatric comorbidities, depressive and cyclothymic temperaments assessed using TEMPS‐A were associated with depressive symptoms at one‐year follow‐up. Depressive temperament was also associated with non‐remission. Also, the association between cyclothymic temperament and one‐year depressive symptom severity differed between MDD and BD. These findings suggest that affective temperaments may be clinically relevant for understanding subsequent depressive symptoms after a major depressive episode in a clinically‐defined sample with a restricted age range and without clinically relevant psychiatric comorbidities. Larger prospective studies are required to confirm these findings and to clarify the clinical utility of affective temperament assessment in mood disorders.

## Author Contributions

T.O. collected and analyzed the data and drafted the manuscript. H.N., S.M., N.U., N.M., N.H., I.K., and T.A.K. supervised the entire process of data acquisition and manuscript writing. All authors reviewed the final version of the manuscript and approved its submission.

## Funding

The authors report no financial or other relationships that are relevant to the subject of this article. I.K. has received honoraria from AbbVie, Boehringer Ingelheim, Daiichi‐Sankyo, Eisai, Janssen Pharmaceutical, Kowa AG Pharma, Meiji Seika Pharma, MSD, Novartis Pharma, Otsuka Pharmaceutical, Shionogi, Sumitomo Pharma, Takeda Pharmaceutical, Teijin Pharma, and Viatris, and has received research/grant support from Sumitomo Pharma. T.O., H.N., S.M., N.U., N.M., N.H., and T.A.K. have no potential conflicts of interest.

## Ethics Statement

This study was approved by the ethics committee of the Hokkaido University Graduate School of Medicine.

## Consent

Written informed consent was obtained from all participants after they had received a full explanation of the study procedures.

## Conflicts of Interest

The authors report no financial or other relationships that are relevant to the subject of this article. T.A.K. has received honoraria from Daiichi‐Sankyo Pharma, Eisai Pharma, Meiji Seika Pharma, Mochida Pharma, MSD Pharma, Nobel Pharma, Ono Pharma, Otsuka Pharma, Shionogi Pharma, Sumitomo Pharma, Takeda Pharma, Tanabe‐Mitsubishi Pharma, and Viatris Pharma. I.K. has received honoraria from AbbVie, Boehringer Ingelheim, Daiichi‐Sankyo, Eisai, Janssen Pharmaceutical, Kowa AG Pharma, Meiji Seika Pharma, Mitsubishi‐Tanabe Pharma, MSD, Novartis Pharma, Otsuka Pharmaceutical, Shionogi, Sumitomo Pharma, Takeda Pharmaceutical, Teijin Pharma, Tsumura, and Viatris, and has received research/grant support from Eisai, Mitsubishi Tanabe Pharma, Otsuka Pharmaceutical, Shionogi, Sumitomo Pharma, and Takeda Pharmaceutical. T.O., H.N., S.M., N.U., N.M., and N.H. have no potential conflicts of interest.

## Supporting information


**Table S1:** Spearman correlations among TEMPS‐A temperament scores.
**Table S2:** Associations between TEMPS‐A temperaments and HAM‐D17 suicidal ideation item score at one‐year follow‐up.
**Table S3:** Associations between bipolar‐spectrum features and depressive symptoms at one‐year follow‐up.
**Table S4:** Sensitivity analysis of the associations between affective temperaments and depressive symptoms at one‐year follow‐up using the final diagnosis.
**Table S5:** Sensitivity analysis of temperament‐by‐diagnosis interactions in relation to depressive symptoms at one‐year follow‐up using the final diagnosis.
**Figure S1:** Interaction between cyclothymic temperament and diagnostic group in relation to depressive symptoms at one‐year follow‐up. Adjusted predicted standardized HAM‐D17 total scores at one‐year follow‐up are shown according to standardized cyclothymic temperament scores for patients with major depressive disorder (MDD) and bipolar disorder (BD). Shaded areas indicate 95% confidence intervals. The cyclothymic temperament‐by‐diagnostic group interaction was significant (standardized *β* = 0.45, 95% CI = 0.09–0.82, *p* = 0.015).

## Data Availability

The datasets generated and analyzed during the current study are not publicly available because they contain sensitive clinical data from human participants, and public data sharing was not covered by the original informed consent procedures. De‐identified data may be available from the corresponding author on reasonable request, subject to approval by the relevant ethics committee.
