# Supplementary material for: Association of Affective Temperaments With One‐Year Depressive Outcomes in Middle‐Aged and Older Patients With Mood Disorders Without Psychiatric Comorbidities
Source: Neuropsychopharmacol Rep. 2026 Sep 16;46(3):e70170. doi: 10.1002/npr2.70170 (PMC13581677; doi:10.1002/npr2.70170)
Supplement: Supplementary file 1 [file NPR2-46-e70170-s001.docx]

**Supporting Information**

**Table S1. Spearman correlations among TEMPS-A temperament scores**

| TEMPS-A |  | Depressive | Cyclothymic | Hyperthymic | Irritable | Anxious |
| --- | --- | --- | --- | --- | --- | --- |
| Depressive |  | — |  |  |  |  |
| Cyclothymic |  | 0.55** | — |  |  |  |
| Hyperthymic |  | -0.11 | 0.19 | — |  |  |
| Irritable |  | 0.51** | 0.62** | 0.03 | — |  |
| Anxious |  | 0.71** | 0.73** | -0.02 | 0.61** | — |

Values are Spearman’s rank correlation coefficients (ρ).

**p < 0.001.

Abbreviations: TEMPS-A, Temperament Evaluation of Memphis, Pisa, Paris, and San Diego Autoquestionnaire.

**Table S2. Associations between TEMPS-A temperaments and HAM-D17 suicidal ideation item score at one-year follow-up**

| **Variables** |  | **β** | **95% CI** | **p value** |  | **Adjusted R^2^** |
| --- | --- | --- | --- | --- | --- | --- |
| Depressive temperament |  | 0.18 | [-0.05, 0.39] | 0.106 |  | 0.16 |
| Cyclothymic temperament |  | 0.27 | [0.09, 0.50] | 0.015 | * | 0.19 |
| Hyperthymic temperament |  | 0.04 | [-0.13, 0.29] | 0.692 |  | 0.14 |
| Irritable temperament |  | 0.27 | [0.04, 0.45] | 0.018 | * | 0.19 |
| Anxious temperament |  | 0.27 | [0.02, 0.44] | 0.026 | * | 0.19 |

Multiple linear regression analyses were performed to examine associations between TEMPS-A affective temperament scores and the suicidal ideation item of the HAM-D17 at one-year follow-up. Each temperament score was entered separately into the model. Models were adjusted for age, sex, diagnostic group, baseline HAM-D17 total score, and psychotropic medication use. β indicates the standardized regression coefficient. Standardized β coefficients and corresponding 95% confidence intervals were presented.

Abbreviations: TEMPS-A, Temperament Evaluation of Memphis, Pisa, Paris, and San Diego Autoquestionnaire; HAM-D17, 17-item Hamilton Depression Rating Scale; CI, confidence interval.

**Table S3. Associations between bipolar-spectrum features and depressive symptoms at one-year follow-up**

| **Variable** |  | **β** | **95% CI** | **p value** |  | **Adjusted R^2^** |
| --- | --- | --- | --- | --- | --- | --- |
| Recurrent MDEs |  | 0.18 | [-0.24, 0.60] | 0.390 |  | 0.25 |
| Early age at onset of first MDE |  | -0.40 | [-1.00, 0.30] | 0.262 |  | 0.25 |
| Treatment-resistant depression |  | 0.01 | [-0.40, 0.42] | 0.961 |  | 0.24 |
| Antidepressant-induced hypomania or mania |  | 0.42 | [-0.02, 0.86] | 0.061 |  | 0.27 |
| Mixed features according to Benazzi's criteria |  | -0.21 | [-0.80, 0.38] | 0.480 |  | 0.25 |
| YMRS total score at baseline |  | 0.03 | [-0.17, 0.23] | 0.779 |  | 0.24 |

Multiple regression analyses were performed to examine associations between bipolar-spectrum features and the HAM-D17 total score at one-year follow-up. Each bipolar-spectrum feature was entered separately into the model. Models were adjusted for age, sex, diagnostic group, baseline HAM-D17 total score, and psychotropic medication use, including antidepressants, mood stabilizers, antipsychotics, and benzodiazepines. For binary predictors, coefficients represent the adjusted difference in standardized one-year HAM-D17 total score between participants with and without the corresponding feature.

Abbreviations: HAM-D17, 17-item Hamilton Depression Rating Scale; YMRS, Young Mania Rating Scale; MDE, major depressive episode; CI, confidence interval, * p < 0.05

**Table S4. Sensitivity analysis of the associations between affective temperaments and depressive symptoms at one-year follow-up using the final diagnosis**

| **Variables** |  | **β** | **95% CI** | **p value** |  | **Adjusted R^2^** |
| --- | --- | --- | --- | --- | --- | --- |
| Depressive temperament |  | 0.21 | [0.02, 0.41] | 0.034 | * | 0.29 |
| Cyclothymic temperament |  | 0.23 | [0.04, 0.42] | 0.018 | * | 0.30 |
| Hyperthymic temperament |  | 0.16 | [-0.03, 0.35] | 0.089 |  | 0.27 |
| Irritable temperament |  | 0.13 | [-0.06, 0.33] | 0.172 |  | 0.26 |
| Anxious temperament |  | 0.20 | [-0.01, 0.41] | 0.056 |  | 0.28 |

As a sensitivity analysis, five patients who were subsequently diagnosed with BD according to their final diagnosis were reclassified. Separate multiple linear regression models were fitted for each TEMPS-A temperament.

Abbreviations: MDD, major depressive disorder; BD, bipolar disorder; TEMPS-A, Temperament Evaluation of Memphis, Pisa, Paris, and San Diego Autoquestionnaire; HAM-D17, 17-item Hamilton Depression Rating Scale; CI, confidence interval.

**Table S5. Sensitivity analysis of temperament-by-diagnosis interactions in relation to depressive symptoms at one-year follow-up using the final diagnosis**

|  |  |  |  | **HAM-D17 total score at one-year follow-up** | | | | |
| --- | --- | --- | --- | --- | --- | --- | --- | --- |
| **Variables** | |  |  | **β** | **95% CI** | **p value** |  | **Adjusted R^2^** |
|  | Depressive temperament | |  |  |  |  |  |  |
|  |  | TEMPS-A (Depressive) |  | 0.20 | [-0.09, 0.48] | 0.177 |  | 0.28 |
|  |  | Diagnosis (BD vs MDD) |  | -0.17 | [-0.62, 0.28] | 0.463 |  |  |
|  |  | TEMPS-A x Diagnosis |  | 0.03 | [-0.34, 0.40] | 0.877 |  |  |
|  | Cyclothymic temperament | |  |  |  |  |  | 0.33 |
|  |  | TEMPS-A (Cyclothymic) |  | -0.07 | [-0.40, 0.25] | 0.662 |  |  |
|  |  | Diagnosis (BD vs MDD) |  | -0.23 | [-0.67, 0.21] | 0.301 |  |  |
|  |  | TEMPS-A x Diagnosis |  | 0.43 | [0.04, 0.81] | 0.029 | * |  |
|  | Hyperthymic temperament | |  |  |  |  |  | 0.27 |
|  |  | TEMPS-A (Hyperthymic) |  | 0.28 | [-0.06, 0.61] | 0.102 |  |  |
|  |  | Diagnosis (BD vs MDD) |  | -0.16 | [-0.61, 0.29] | 0.483 |  |  |
|  |  | TEMPS-A x Diagnosis |  | -0.17 | [-0.57, 0.24] | 0.413 |  |  |
|  | Irritable temperament | |  |  |  |  |  | 0.26 |
|  |  | TEMPS-A (Irritable) |  | -0.01 | [-0.38, 0.35] | 0.940 |  |  |
|  |  | Diagnosis (BD vs MDD) |  | -0.17 | [-0.62, 0.29] | 0.473 |  |  |
|  |  | TEMPS-A x Diagnosis |  | 0.20 | [-0.22, 0.62] | 0.341 |  |  |
|  | Anxious temperament | |  |  |  |  |  |  |
|  |  | TEMPS-A (Anxious) |  | 0.07 | [-0.26, 0.39] | 0.683 |  | 0.28 |
|  |  | Diagnosis (BD vs MDD) |  | -0.16 | [-0.61, 0.29] | 0.473 |  |  |
|  |  | TEMPS-A x Diagnosis |  | 0.21 | [-0.17, 0.59] | 0.276 |  |  |

As a sensitivity analysis, five patients who were subsequently diagnosed with BD according to their final diagnosis were reclassified. Multiple linear regression analyses were performed using standardized TEMPS-A temperament scores and standardized HAM-D17 total score at one-year follow-up. MDD was used as the reference category for diagnosis. The TEMPS-A coefficient represents the association between each standardized temperament score and the standardized HAM-D17 total score in the MDD group. The TEMPS-A × diagnosis coefficient represents the difference in the TEMPS-A slope between BD and MDD.

Abbreviations: MDD, major depressive disorder; BD, bipolar disorder; TEMPS-A, Temperament Evaluation of Memphis, Pisa, Paris, and San Diego Autoquestionnaire; HAM-D17, 17-item Hamilton Depression Rating Scale; CI, confidence interval, β: Standardized β

**Figure S1. Interaction between cyclothymic temperament and diagnostic group in relation to depressive symptoms at one-year follow-up.** Adjusted predicted standardized HAM-D17 total scores at one-year follow-up are shown according to standardized cyclothymic temperament scores for patients with major depressive disorder (MDD) and bipolar disorder (BD). Shaded areas indicate 95% confidence intervals. The cyclothymic temperament-by-diagnostic group interaction was significant (standardized β = 0.45, 95% CI = 0.09–0.82, p = 0.015).
